# Modified Arnold expander: an alternative for mandibular arch expansion

**DOI:** 10.1590/2177-6709.26.5.e21spe5

**Published:** 2021-10-25

**Authors:** Ildeu ANDRADE, Marco Aurélio Benini PASCHOAL, Natália Couto FIGUEIREDO

**Affiliations:** 1 Medical University of South Carolina, Department of Orthodontics (Charleston/SC, USA).; 2 Universidade Federal de Minas Gerais, Departamento de Saúde Bucal da Criança e do Adolescente (Belo Horizonte/MG, Brazil).; 3 Pontifícia Universidade Católica de Minas Gerais Departamento de Ortodontia, (Belo Horizonte/MG, Brazil).

**Keywords:** Malocclusion, Orthodontics, interceptive, Palatal expansion technique, Tooth crowding, Case reports

## Abstract

**Introduction:**

Due to the anatomical constraints of the mandible, mandibular dental arch usually serves as a guideline to determine the required changes in the maxillary transverse dimension. The Schwarz appliance and the Lip Bumper are the traditional orthodontic appliances for mandibular arch expansion in patients with borderline amounts of crowding, and/or transverse discrepancy. However, they often require patient cooperation, which may be a concern for orthodontists in daily practice.

**Objectives:**

This article illustrates a simple fixed orthodontic device as an alternative to achieve mandibular arch expansion in patients with moderate tooth-size/arch-length discrepancy. The four reported cases refer to 8 to 10-year-old patients in the mixed dentition, with an Angle Class I or Class II malocclusion, transverse deficiency in both arches, moderate crowding and/or posterior crossbite, combined with compromised smile aesthetics. The patients were treated with rapid maxillary expansion (RME) using Hass expander appliance and the modified Arnold expander (MAE).

**Conclusion:**

This low-cost compliance-free orthodontic appliance provided dentoalveolar decompensation by means of uprighting the posterior teeth, with minimal or no adjustments during treatment. The final results were achieved in only three to four months, and fulfilled all treatment objectives, such as an increase in the arch perimeter and width, and a better teeth alignment.

## RESUMO

**Introdução:**

Devido aos limites anatômicos da mandíbula, a arcada dentária inferior geralmente serve como guia para determinar as alterações necessárias na dimensão transversal da maxila. O aparelho de Schwarz e o Lip Bumper são os aparelhos usados tradicionalmente para expansão da arcada inferior em pacientes com quantidades limítrofes de apinhamento e/ou discrepância transversal. No entanto, eles requerem a cooperação do paciente, o que pode ser uma preocupação para os ortodontistas na prática diária.

**Objetivos:**

O presente artigo ilustra uma alternativa diferente de aparelho fixo para se obter a expansão da arcada inferior em pacientes com discrepância moderada de tamanho dentário e/ou comprimento da arcada. Os quatro casos relatados referem-se a pacientes com 8 a 10 anos de idade, na dentição mista, com má oclusão de Classe I ou II de Angle, deficiência transversal em ambas as arcadas, apinhamento moderado e/ou mordida cruzada posterior, apresentando comprometimento da estética do sorriso. Os pacientes foram tratados com expansão rápida da maxila (ERM), usando aparelho expansor de Hass, e expansor Arnold modificado (EAM).

**Conclusão:**

O EAM, que é um aparelho de baixo custo e não depende da colaboração do paciente, promoveu uma descompensação dentoalveolar por meio da verticalização dos dentes posteriores, necessitando de mínimo ou nenhum ajuste durante o tratamento. Os resultados pretendidos foram alcançados em três a quatro meses e cumpriram todos os objetivos do tratamento, como aumento do perímetro e largura da arcada, assim como o melhor alinhamento dos dentes.

## INTRODUCTION

The transverse dimension and shape of both dental arches varies widely between individuals, according to dental alignment, tooth shape and size, musculature, jaw size and shape, facial and cranial patterns and the dental occlusion.^1^ The transverse discrepancy between the maxillary and mandibular arches is one of the most commonly seen malocclusions in the primary and mixed-dentition stages.^2^ The prevalence of posterior crossbite is 14% in the primary dentition and 8% in the mixed dentition.^3^ These patients may present narrow posterior transarch widths, related crowding, wide buccal corridors, and decreased anterior arch contour.^4^ However, although the constriction of the jaw bones is frequently associated to posterior crossbite, this is not a mandatory condition, considering that the maxilla and mandible can be dentoskeletal compensated in order to maintain jaw relationships with function,^5^ ^,^ ^6^ In other words, patients without posterior crossbites can have significant transverse discrepancies that might need treatment.

### EFFECTS OF RAPID MAXILLARY EXPANSION (RME)

The transverse malocclusions do not self-correct without treatment, and the expansion of one or both arches is widely recommended, especially during the mixed-dentition period.^7^ ^,^ ^8^ The ideal goal of RME is to achieve minimal dental and maximum orthopedic effect.^9^ Different studies have reported that it affects the circummaxillary sutures, specifically the midpalatal one, compresses the periodontal ligament, bends the alveolar processes and induces a buccal tipping of the anchoring teeth, among other skeletal and dental effects.^10^ ^-^ ^12^ The transverse expansion will result in varied intra-arch dimensional changes, in addition to potentially altering the occlusal relationships in the three planes of space. It has been shown that RME therapy can increase the maxillary arch perimeter by 0.7 mm for every millimeter of posterior expansion^7^ ^,^ ^12^. However, it is noteworthy that the amount of expansion created by a given RME protocol is variable and relies on the goals of the orthodontist. As an example, Haas recommends opening the expander to the full extent of the screw (10.0 to 10.5 mm), thereby maximizing the increase in arch width.^7^ ^,^ ^9^ ^,^ ^10^ Other study^13^ demonstrated that patients who were treated with RME during the mixed-dentition phase followed by fixed appliances had a maxillary arch perimeter 2.7 mm larger and a mandibular arch perimeter 2.0 mm larger, in comparison to non-treated patients (by spontaneous mandibular intermolar expansion). 

### MANDIBULAR EXPANSION AND WALA RIDGE

In order to correct these transverse deficiencies and maximize the RME, the mandibular expansion can be a meaningful tool, particularly in cases of mild to moderate discrepancy between tooth size and arch lenght.^7^ However, gaining space in the mandibular arch has been considered as a limiting factor, by anatomic reasons and due to the belief that the expansion is not stable. Housley et al.^14^ demonstrated that an increase in mandibular arch width of 1.52mm in permanent canines, 2.11mm in first premolars, 2.12mm in second premolars, and 0.92mm in permanent first molars, carried out with an expanding lingual arch appliance, relapsed in 0.8mm, 0.72mm, 0.67mm and 0.15mm, respectively, after a mean postretention period of 6 years and 3 months (? 2 years and 4 months). Nevertheless, the mean pretreatment age in this study was 12 years and 5 months, and most patients were in permanent dentition. Despite the noted relapse effect, particularly in the anterior arch region, it can be speculated that the transverse expansion performed in the deciduous or early mixed dentition may present a different behavior. Early widening of the dental arches might positively influence the subsequent growth and development of bone jaws, besides a favorable adaption of the muscular environment, which can alter the eruptive paths of the permanent teeth in a buccal direction.^15^

**Figure 1:**
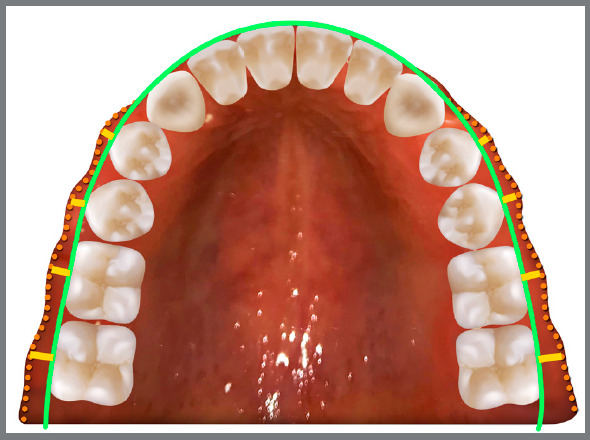
Representation of the individualized dental arch form (green line) according to the WALA ridge (orange dotted lines). The yellow lines represent the average distances between the facial axis-WALA ridge, which is gradually increased in the posterior sites.

Furthermore, it has been reported that the mandibular arch form has a correlation to the shape of the underlying basal bone, which can potentially be used as a reliable diagnostic reference for determining the best position of the mandibular teeth, providing a more stable orthodontic treatment outcome.^1^ ^,^ ^16^ With that purpose, Andrews and Andrews^17^ proposed the WALA ridge as an anatomic reference on the mandibular alveolar process that demarcated the soft-tissue band immediately superior to the mucogingival junction,^18^ which is located close to the same vertical level as the horizontal center of rotation of each tooth.^19^ The WALA ridge is easy to identify and might be clinically useful for individualizing dental arch shape^20^ (Fig 1). 

Typical treatment protocols for patients needing mandibular expansion are the removable mandibular Schwarz appliance^21^ and the Lip Bumper.^22^ The protocol may begin with maxillary expansion or mandibular decompensation. The Schwarz appliance, which is usually activated once a week for approximately 5 to 6 months, provides a dentoalveolar decompensation of the mandibular arch, establishing a “reference” arch width to which the maxillary arch can be expanded.^23^ Later on, the Schwarz appliance should be worn full-time as a passive retainer until the maxillary expander is removed. The primary purpose of the Lip Bumper is to reduce dental arch crowding ^24^ through an increase in arch width and length,^25^ ^,^ ^26^ by altering the equilibrium between lips, cheeks, and tongue.^27^ ^,^ ^28^ However, as removable appliances, the expansion rate is slow, due to problems with retention and compliance, which might be an important clinical drawback.^29^ ^,^ ^30^

### MODIFIED ARNOLD EXPANDER (MAE)

**Figure 2:**
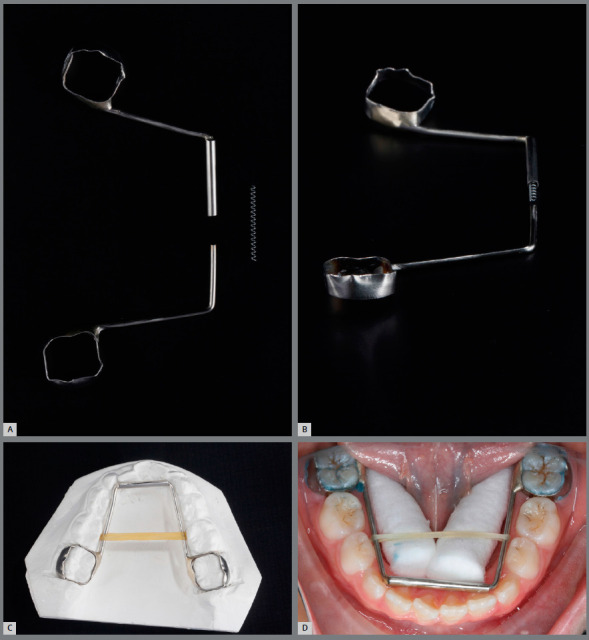
Modified Arnold expander. A) Component parts of the appliance. B) Nickel-titanium open-coil spring inside the tube, before connecting the appliance’s parts. **C, D**) Inserted device, with elastic holding the two segments together.

An interesting device to overcome these issues is the Arnold expander, which became popular in the 1970s by Berkowitz^31^ as a way to produce slow expansion of the maxillary or mandibular arches, especially in cleft-palate patients, as a non-compliance alternative solution for the correction of tooth size/arch length discrepancy.^32^ However, its asymmetric expansion, difficulties of cleaning the exposed open coil, and common tongue injuries have discouraged the use of this appliance. Thus, in the present article, four cases treated in a private office will be presented, in which a modified Arnold expander (MAE) overcame these issues and promoted an increase in the transverse dimension of the mandibular arch in a quick and cheap way. This device has a split lingual frame, a 0.040-in stainless steel tube that was welded to the lingual side of the permanent first molar band and a 0.038-in stainless steel wire welded to the opposite molar band. In both sides, the structure runs lingual to the deciduous molars and canines, and turns at a 90^o^ angle at the midpoint of the canine. The two parts fit together, with the wire sliding through the tube at the midline, like a telescopic system (Fig 2). A nickel-titanium (NiTi) open-coil spring (0.010 x 0.030-in, G&H Orthodontics, Franklin, IN, USA) is interposed among the two parts inside of the tube (Fig 2B). Seating the device compresses the coil spring and activates it for expansion.

### CASE REPORTS

#### CASE 1

**Figure 3:**
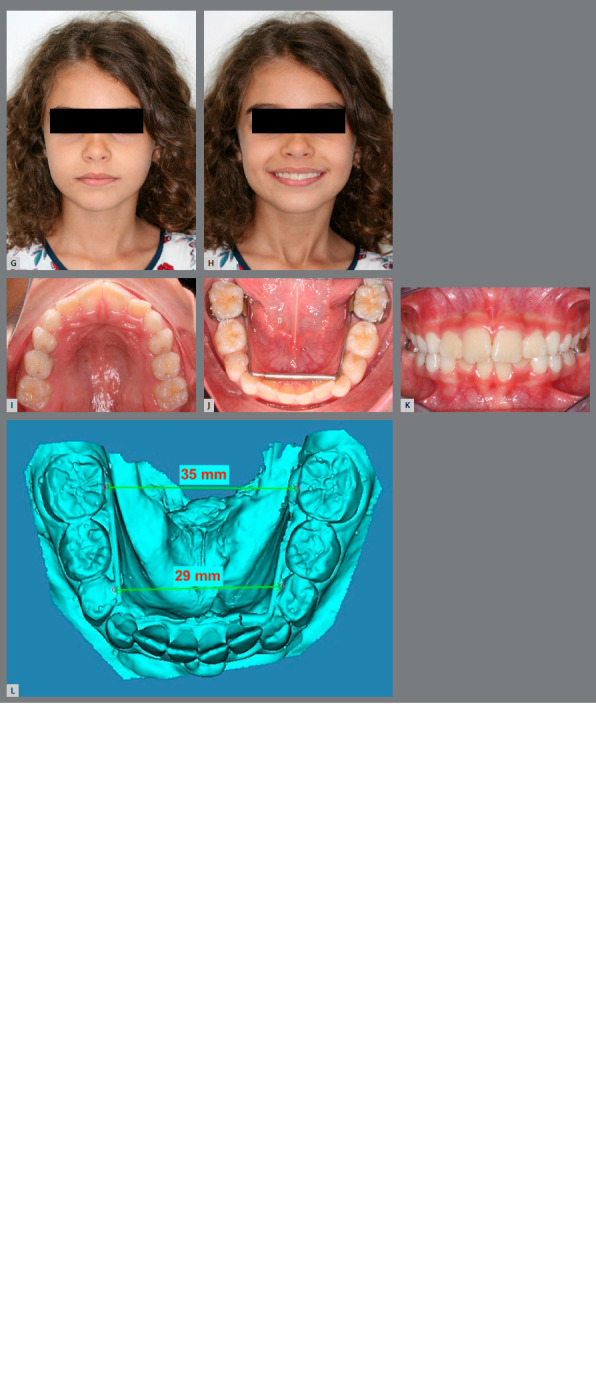
A, B) Pretreatment facial photographs. C-E) Pretreatment intraoral photographs. F) Pretreatment mandibular plaster model, scanned using the Smart Optical 3D Scanner^®^ (Open Technologies, Rezzato, Italy), showing an intermolar width of 30 mm between the permanent first molars and 22 mm between the deciduous first molars. G-H) Post-treatment facial photographs. I-K) Post-treatment intraoral photographs. L) Post-treatment 3D model of the mandibular arch, showing an intermolar width of 35 mm between the permanent first molars and 29 mm between the deciduous first molars.

A healthy 8-year-old girl, with a chief complaint of crowding, was referred to the office for orthodontic evaluation. There was no history of dental trauma or oral habits. Pretreatment facial analysis revealed a symmetrical face, proper vertical ratio, competent lips, and a slightly convex profile (Figs 3A and 3B). Her smile aesthetics was compromised due to the greater exposure of mandibular teeth and asymmetric buccal corridors. The intra-oral examination revealed a Class I molar relationship on both sides. The patient was at the beginning of the mixed dentition, presenting a poor prognosis for the eruption of the maxillary and mandibular lateral incisors. Both arches were constricted, with a deep palatal vault and a moderate negative space discrepancy (Figs 3C - 3F). 

The orthodontic treatment objectives for the first phase were to: (1) increase the transverse dimension of both arches; (2) resolve crowding and obtain space for the alignment of permanent teeth; (3) improve the smile aesthetics; and (4) maintain the facial balance. The treatment plan included a RME with a Haas expander appliance, combined with a mandibular expansion by means of a MAE.

After four months of treatment, there was a noticeable increase in the lip intercommissural distance and a significant improvement in smile aesthetics, due to the increased maxillary incisors display and harmonic buccal corridors (Figs 3G - 3H). A better arch form, with an increase in the arch perimeter, was achieved (Figs 3I - 3L). The Haas expander was placed on the deciduous second molars and activated with a ¼ turn once a day, during 20 days, until 4 and 5 mm of transversal expansion was obtained in the intermolar and intercanine width, respectively. The MAE was installed in the mandibular permanent first molars (Fig 3J). To facilitate the insertion, an ¼-in orthodontic elastic should join the two sections together during installation (Figs 2C - 2D). To avoid displacement of the anterior part during treatment and promote stability of segments, both sides can be bonded to deciduous canines or deciduous first molars with flowable composite. 

In the occlusal view, the gain in intercanine and intermolar widths (5 mm between the permanent mandibular first molars and 7 mm between the deciduous mandibular first molars) was remarkable in both arches, with a considerable uprighting of the mandibular molars in the transverse plane (Figs 3D, 3F, 3J, 3L). When comparing the facial parameters, treatment objectives were achieved, with excellent esthetic and functional results. 

#### CASE 2

**Figure 4:**
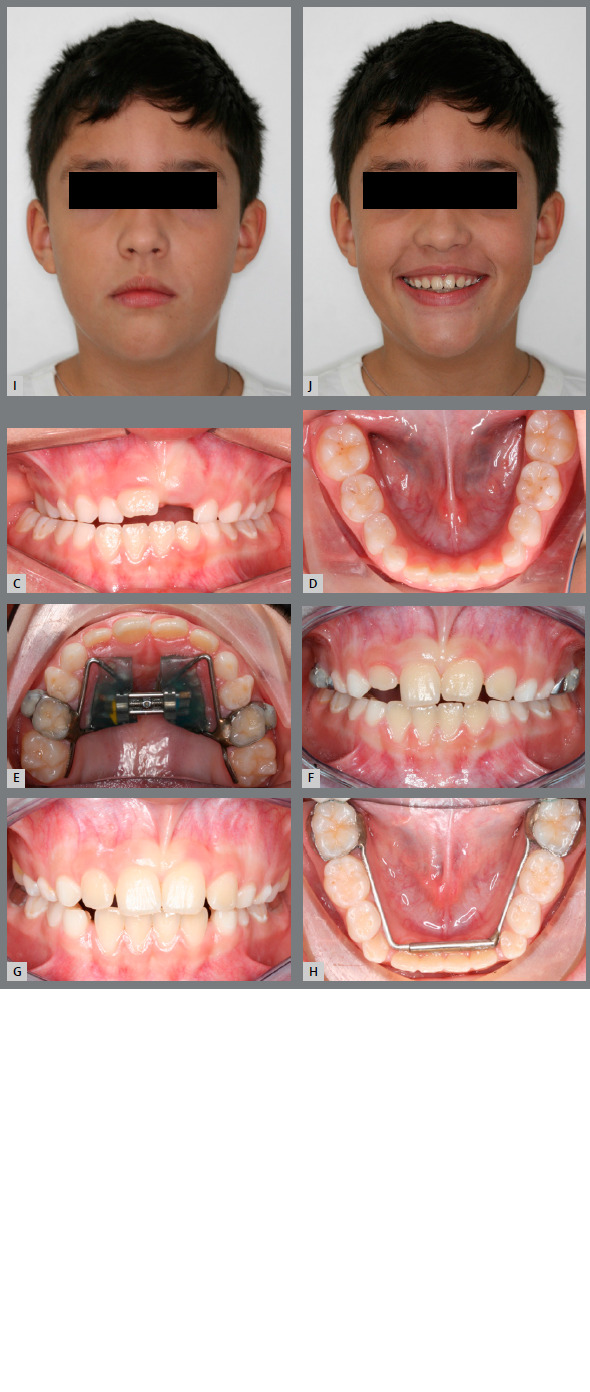
A,B) Pretreatment facial photographs. C,D) Pretreatment intraoral photographs. E,F) Progress intraoral photographs, showing the posterior crossbite correction with the Haas expander appliance. G,H) Post-treatment intraoral photographs. A 2-mm space was gained on both sides of the mandibular arch due to the MAE. I, J) Post-treatment facial photographs, showing significant improvement in smile esthetics. It is possible to notice greater exposure of the maxillary incisors and harmonic buccal corridors.

An 8-year-old boy, with the chief complaint of chewing impairment, was referred to orthodontic treatment. He was in good general health, with no systemic or congenital disease. Pretreatment facial analysis revealed a slightly convex profile, a mild facial asymmetry, with a mandibular deviation of 3 mm to the right, and lip competence. The patient was at the beginning of a mixed dentition phase, with an Angle Class I malocclusion, unilateral posterior crossbite, and transverse deficiency in both arches (Figs 4A - 4D). 

Hence, the aims of the treatment were to expand the maxillary and mandibular arches in order to gain space, correct the posterior crossbite, and obtain normal overjet, overbite, and incisor inclinations. The RME was performed with a Haas expander appliance. The parents were advised to activate the screw by ¼ turn per day during 28 days, when an excellent orthopedic response was verified (increase of 5 mm in the intermolar width, and 6 mm in the intercanine width), resulting in the crossbite correction (Figs 4E and 4F). Later on, the MAE was inserted, which was maintained in place for four months. At the end of this period, a significant space gain in arch perimeter (2 mm on both sides of both dental arches) could be observed in the post-treatment photographs (Figs 4G and 4H). After four months of treatment, there was a significant improvement in smile aesthetics due to the increased maxillary incisors display and harmonic buccal corridors (Figs 4I and 4J). Upon conclusion of the first phase of orthodontic treatment, an Angle Class I molar relationship was maintained, with normal overjet and overbite. Moreover, the appliances promoted space gain that will accommodate the permanent teeth and provide good dental intercuspation, with the correction of the posterior crossbite. The treatment resulted in significant improvements in dental alignment and smile.

#### CASE 3

**Figure 5:**
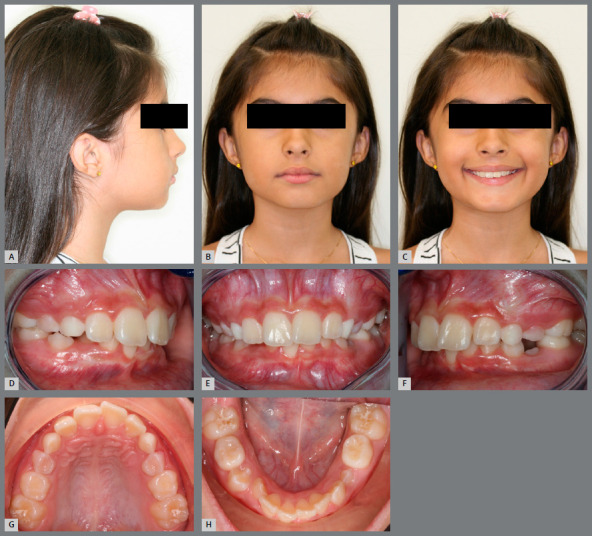
Pretreatment photographs: **A-C**) facial photographs; **D-H**) intraoral photographs.

A 10-year-old girl, seeking orthodontic treatment for correction of a skeletal Class II malocclusion characterized by a deep overbite that was associated to a mandibular right central incisor trauma, which was causing a gingival recession. Pretreatment examination revealed a late mixed dentition stage of development, with moderate mandibular incisor crowding and arch length deficiency in the maxillary arch (Figs 5A - 5H). 

The molar relationship was full-cusp Class II on the left side and end-to-end Class II on the right. The maxillary incisors were protruded, and the mandibular ones were somewhat upright in appearance. A severe deep overbite of approximately 6 - 7 mm was noted and the overjet was measured at 5 mm. The maxillary midline was deviated to the right.

The profile photograph shows a convex profile with normal facial thirds. Lip line, upper lip length, and nasolabial angle were all considered normal. Skeletal analysis, as obtained by cephalometry, shows a skeletal Class II facial pattern. The treatment objectives for this first phase treatment were as follows: (1) Achieve a Class I molar relationship; (2) gain space in the maxillary arch for the canines and for the correction of the maxillary incisor proclination; (3) eliminate the lower incisor trauma; and (4) reduce skeletal disharmony to improve the facial profile.

**Figure 6:**
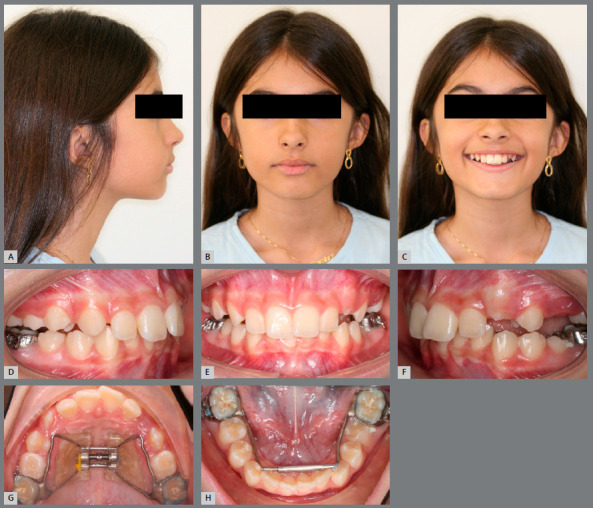
A-H) Progress records. D-E) Progress intra-oral photographs, showing the Haas expander and the Arnold expander appliances.

In order to achieve these objectives, a RME was promoted by means of a Hass palatal expander, followed by a MAE in the mandibular arch, in order to coordinate the transverse dimensions of the maxillary and mandibular arches (Fig 6). Furthermore, a cervical pull headgear was proposed to correct the sagittal discrepancy. 

**Figure 7:**
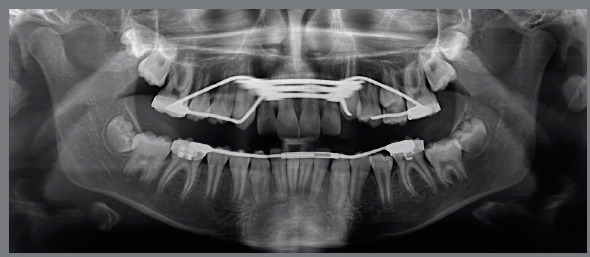
Progress panoramic radiograph.

As instructed, the parents activated the screw by two turns per day during 10 days, when an excellent orthopedic response was verified (5-mm diastema between central incisors, with an increase of 4 mm in the intermolar width and 4.5 mm in the intercanine width). The MAE was kept in place for four months. At the end of this period, a significant improvement in the arch perimeter (4 mm on the maxillary arch and 3 mm in the mandibular arch) and width was obtained, as can be observed in the post-treatment photographs (Figs 6G and 6H). After four months of treatment, there was plenty of space for the maxillary canines (Fig 7), the mandibular incisor trauma was eliminated, and the gingival recession was improved (Fig 6E).

#### CASE 4

**Figure 8:**
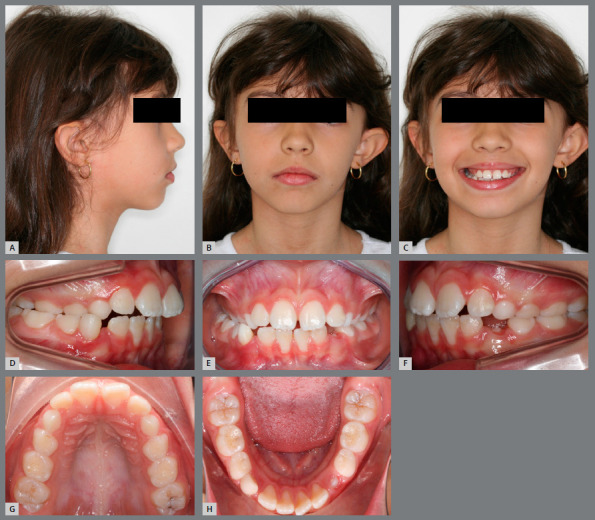
Pretreatment photographs: **A-C**) facial photographs; **D-H**) intraoral photographs.

A 9 year-old girl, with unilateral crossbite and lower midline deviation to the right side, sought orthodontic treatment. Pretreatment facial analysis revealed a slightly convex profile, a mild facial asymmetry, with a 2-mm mandibular deviation to the right side, and lip competence (Figs 8A - 8C). The patient was in the mixed dentition phase, with an Angle Class II malocclusion, unilateral posterior crossbite and transverse deficiency in both dental arches (Figs 8D - 8H). 

**Figure 9:**
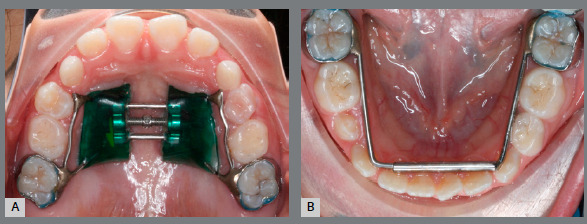
A-B) Progress photos of both arches.

**Figure 10:**
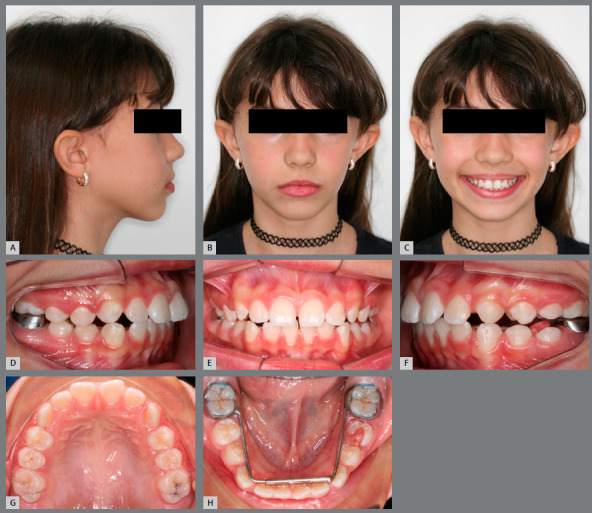
Post-treatment photographs.

The treatment plan for this first phase included the decompensation of the mandibular arch at the same time of the maxillary expansion. The MAE was placed first, and the Haas expander in the following month. The parents were advised to activate the screw by ¼-turn twice a day during two weeks, and then ¼-turn per day for a week, when an excellent orthopedic response was verified (diastema between central incisors of 5mm, with an increase of 4 mm in the intermolar width and 5 mm in the intercanine width), resulting in an increase in the transverse dimension of both arches and, consequently, correction of the crossbite (Fig 9). After five months of treatment, there was a significant improvement in smile aesthetics due to the increased maxillary incisors display and harmonic buccal corridors (Fig 10C). An Angle Class II molar relationship still persisted in both sides, but a significant improvement was noted in the right side. The MAE promoted space gain that would, together with the Leeway space, accommodate the permanent teeth and could be used to mesialize the mandibular posterior teeth to achieve Class I relationship (Figs 10D -10H). 

## DISCUSSION

The size and shape of the dental arches have an important effect on space available, stability of the dentition, and dental esthetics.^19^ Although most orthodontic treatment for transversal discrepancies focuses on the maxilla, it is important to recognize that dental compensations exist for both dental arches. Therefore, the orthodontist must be able to diagnose differentially the cause of any transverse discrepancy, and the presence or absence of posterior crossbite should not be used as a major and unique guide.^33^

The correction of crowding in cases with tooth-size/dental arch length discrepancy might be a key factor when deciding between extraction and nonextraction orthodontic treatment. In order to achieve that, RME is often used during treatment, but mandibular arch widening has primarily been limited to uprighting of posterior teeth, since there is no midline suture, as in the maxilla.^32^ ^,^ ^34^ O’Grady et al.^21^ have reported that the RME-only protocol showed modest long-term increases in maxillary (2.6 mm) and mandibular arch perimeter (2.0 mm), with the latter not being statistically significant. Meanwhile, significant increases in the maxillary (3.8 mm) and mandibular (3.7 mm) arch perimeters were observed when the mandibular arch was also expanded with Schwarz appliance, when compared with the matched control group. Other studies have found good clinical outcomes with mandibular expansion with Lip Bumper.^22^ ^,^ ^35^ Previous studies have shown that the greatest arch width gain occurs in the molar and premolar area and the smallest, in the canine area.^35^ Lip bumper studies have shown increased mandibular perimeter of 4 - 5 mm, which was related to arch width^36^ and arch length changes due to incisor proclination and molar distalization.^24^

O’Grady et al.^21^ reported that mandibular Schwarz appliance combined to RME in the mixed dentition followed by comprehensive orthodontic treatment in permanent dentition induced significant increments in mandibular arch width (+2,6 mm for intermolar width and +2,1 mm for intercanine width). In post-retention evaluation, at least 3 years after the phase II treatment, the arch width decreased 0,3mm for intermolar width and 1,3mm for intercanine width, which means that 2,3mm of transversal gain in the molar region and 0,8mm in the canine region was maintained. In the study of Housley et al.,^14^ the patients treated with expanded lingual arch appliances in the maxillary and mandibular arches followed by fixed appliances presented a greater relapse in the intercanine width, which steady decreased posteriorly in the mandibular arch. Other post-retention studies reported decreases in the molar (0.6mm - 1.5mm), premolar (1.2 mm), and canine (0.4mm - 0.9mm) widths,^22^ ^,^ ^37^ which were not clinically relevant. Although mandibular expansion has been discouraged by some authors due to potentially relapse effect,^21^ ^,^ ^38^ it has been showed that when a crowded mandibular arch is expanded before the eruption of the permanent teeth, the path of eruption of the mandibular permanent canines and premolars might be altered to an increased width.^15^ ^,^ ^39^ Moreover, the greatest growth changes in the dentoalveolar area occur during the eruption of permanent teeth.^40^ In this way, it seems reasonable to take advantage of the eruption dynamics to potentially improve the development of the dentoalveolar area.

**Figure 11:**
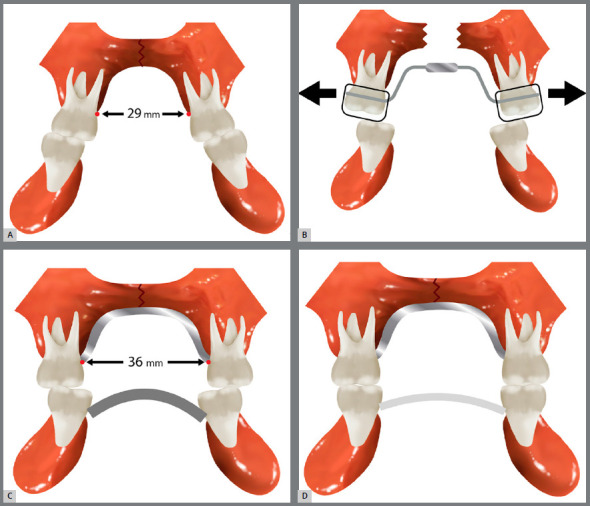
Expansion protocol. A) Maxillary narrow arch, combined with lingual inclination of the mandibular molars, as a compensatory effect. B) Orthopedic maxillary expansion. C) MAE placed in the mandibular arch to decompensate the posterior mandibular teeth. D) Ideal transverse skeletal and dental relationships of both arches, maintained by TPA and lingual arch.

In the first three reported cases, the patients were first submitted to RME, until the lingual cusps of the maxillary posterior teeth contacted the buccal cusps of the mandibular posterior teeth (Figs 11A and 11B). Later on, the MAE was installed and provided a buccolingual decompensation of the posterior teeth and a proper intercuspation (Fig 11C) . Once the ideal transversal relationship was achieved, and after the stabilization period, these appliances were replaced by a transpalatal arch (TPA) and a lingual arch in the maxillary and mandibular arches, respectively (Fig 11D) . 

The patients remained with the TPA and lingual arch as fixed retentions, until the beginning of the second phase of orthodontic treatment. A different protocol was performed in the case 4, with the mandibular expansion being performed previously to the maxillary expansion - which may be a good option, specially when there is no urgency for maxillary expansion. In this way, the new transverse dimension obtained in the mandibular arch may be used as a template for the amount of expansion in the maxilla.

Moreover, with intervention in the early mixed dentition, orthodontists can eliminate potential irregularities and facilitate dental eruption. In permanent dentition, the mandibular expansion is even more controversial.^14^ ^,^ ^41^ Nevertheless, the expansion approach in children and preadolescents can be considered an effective treatment option to gain space in the maxillary and mandibular arches for the management of tooth-size/arch-length discrepancies of mild-to-moderate degree, particularly in patients with transverse deficiency in association with an accentuated curve of Wilson.^42^

The mandibular expansion can create a new reference for the transverse dimension of the maxilla, allowing a greater amount of maxillary expansion.^23^ However, animal studies have shown that predisposing bone dehiscences may be induced by uncontrolled buccal expansion of teeth through the cortical plate, which favors the development of gingival recession.^43^ ^,^ ^44^ Expansion of the arch form has been shown to produce gingival recession when expressed beyond the alveolar bone.^45^ The key for maintaining attachment is to produce movement that results in tooth movement within the alveolar bone, thus preventing any dehiscences. 

The WALA ridge can be used as a template for expansion of both arches, which will match the anterior and lateral borders of the WALA ridge. According to Andrews,^17^ the shape will be uniquely correct for each patient, regardless of race or sex. The WALA ridge may resolve orthodontics’ long-lasting controversies regarding the anterior and lateral arch-border positions and archwire shapes, as well as whether to extract or expand, or both. It also solves the maxillary arch-border and the maxillary width controversies because a uniquely correct mandibular arch’s lateral borders serve as the landmark for the lateral borders for both the upper dental arch and the maxilla. The use of MAE may be a good option in these cases. Regardless, the decision whether to expand or extract requires proper diagnosis, and must be made on a case-by-case basis. In the four cases reported in the present article, the treatment objectives were achieved, with excellent esthetic and functional results. 

Last but not least, it has been shown that parents/caregivers have a determining and critical role in cooperation of their children.^46^ ^,^ ^47^ Moreover, patient compliance may decline due to discomfort such as soft tissue irritation, tooth ache, lack of confidence in public, and speech and respiratory disorders. In the first two cases of this article, the treatment begun with the Schwarz appliance or the Lip Bumper, however the lack of patient-parent cooperation was an issue in the beginning of the treatment. Thus, the MAE was an alternative to removable appliances to correct constricted mandibular arches by posterior teeth uprighting, to relieve crowding, to promote a proper posterior intercuspation and to improve the morphology of the mandibular dental arch without patient cooperation. Furthermore, a fixed mandibular expander offers some advantages, such as a minimal chair-time, no following adjustments, low financial costs, and a symmetrical expansion.

## FINAL CONSIDERATIONS

Taking all together, the use of MAE may be clinically advantageous, since it can be effective in decompensating the mandibular posterior teeth buccally, which allows a greater amount of maxillary expansion that, in turn, can be favorable for increasing arch perimeter of both dental arches. Moreover, the MAE is a low-cost device that do not need adjustments (less chair-time) and patient compliance, and do not present complaints about oral hygiene and/or injuries. An adequate housing of the roots in the bony envelope during the mix dentition stage, and a correct intercuspation upon the end of treatment are important to maintain the final results. Experimental studies are needed to evaluate the effectiveness of the presented protocol, as well as its long-term stability. 

## References

[1] Kong-Zárate CY, Carruitero MJ, Andrews WA (2017). Distances between mandibular posterior teeth and the WALA ridge in Peruvians with normal occlusion. Dental Press J Orthod.

[2] Schindel RH, Duffy SL (2007). Maxillary transverse discrepancies and potentially impacted maxillary canines in mixed-dentition patients. Angle Orthod.

[3] Lombardo G, Vena F, Negri P, Pagano S, Barilotti C, Paglia L (2020). Worldwide prevalence of malocclusion in the different stages of dentition A systematic review and meta-analysis. Eur J Paediatr Dent.

[4] Handelman CS, Balakrishnan M, BeGole EA, Viana GC (2020). Bimaxillary transverse constriction in adults Short-term follow-up of non-surgical arch expansion. Orthod Craniofac Res.

[5] Crossley AM, Campbell PM, Tadlock LP, Schneiderman E, Buschang PH (2020). Is there a relationship between dental crowding and the size of the maxillary or mandibular apical base. Angle Orthod.

[6] Handelman CS, Balakrishnan M, BeGole EA, Viana GC (2020). Bimaxillary transverse constriction in adults Short-term follow-up of non-surgical arch expansion. Orthod Craniofac Res.

[7] McNamara JA (2006). Long-term adaptations to changes in the transverse dimension in children and adolescents an overview. Am J Orthod Dentofacial Orthop.

[8] Bishara SE, Staley RN (1987). Maxillary expansion clinical implications. Am J Orthod Dentofacial Orthop.

[9] Haas AJ (1961). Rapid expansion of the maxillary dental arch and nasal cavity by opening the midpalatal suture. Angle Orthod.

[10] Haas AJ (1970). Palatal expansion just the beginning of dentofacial orthopedics. Am J Orthod.

[11] Cameron CG, Franchi L, Baccetti T, McNamara JA (2002). Long-term effects of rapid maxillary expansion: a posteroanterior cephalometric evaluation. Am J Orthod Dentofacial Orthop.

[12] Ballanti F, Lione R, Fanucci E, Franchi L, Baccetti T, Cozza P (2009). Immediate and post-retention effects of rapid maxillary expansion investigated by computed tomography in growing patients. Angle Orthod.

[13] Geran RG, McNamara JA, Baccetti T, Franchi L, Shapiro LM (2006). A prospective long-term study on the effects of rapid maxillary expansion in the early mixed dentition. Am J Orthod Dentofacial Orthop.

[14] Housley JA, Nanda RS, Currier GF, McCune DE (2003). Stability of transverse expansion in the mandibular arch. Am J Orthod Dentofacial Orthop.

[15] Lutz HD, Poulton DR (1985). Stability of dental arch expansion in the deciduous dentition. Angle Orthod.

[16] Ball RL, Miner RM, Will LA, Arai K (2010). Comparison of dental and apical base arch forms in Class II Division 1 and Class I malocclusions. Am J Orthod Dentofacial Orthop.

[17] Andrews L, Andrews W (2001). The syllabus of the andrews orthodontic philosophy.

[18] Triviño T, Siqueira DF, Andrews WA (2010). Evaluation of distances between the mandibular teeth and the alveolar process in Brazilians with normal occlusion. Am J Orthod Dentofacial Orthop.

[19] Ronay V, Miner RM, Will LA, Arai K (2008). Mandibular arch form the relationship between dental and basal anatomy. Am J Orthod Dentofacial Orthop.

[20] Zou W, Wu J, Jiang J, Xu T, Li C (2014). Archform comparisons between skeletal class II and III malocclusions. PLoS One.

[21] O'Grady PW, McNamara JA, Baccetti T, Franchi L (2006). A long-term evaluation of the mandibular Schwarz appliance and the acrylic splint expander in early mixed dentition patients. Am J Orthod Dentofacial Orthop.

[22] Solomon MJ, English JD, Magness WB, McKee CJ (2006). Long-term stability of lip bumper therapy followed by fixed appliances. Angle Orthod.

[23] McNamara JA (2000). Maxillary transverse deficiency. Am J Orthod Dentofacial Orthop.

[24] Davidovitch M, McInnis D, Lindauer SJ (1997). The effects of lip bumper therapy in the mixed dentition. Am J Orthod Dentofacial Orthop.

[25] Murphy CC, Magness WB, English JD, Frazier-Bowers SA, Salas AM (2003). A longitudinal study of incremental expansion using a mandibular lip bumper. Angle Orthod.

[26] Häsler R, Ingervall B (2000). The effect of a maxillary lip bumper on tooth positions. Eur J Orthod.

[27] O'Donnell S, Nanda RS, Ghosh J (1998). Perioral forces and dental changes resulting from mandibular lip bumper treatment. Am J Orthod Dentofacial Orthop.

[28] Proffit WR (1978). Equilibrium theory revisited factors influencing position of the teeth. Angle Orthod.

[29] Tai K, Park JH, Mishima K, Shin JW (2011). 3-Dimensional cone-beam computed tomography analysis of transverse changes with Schwarz appliances on both jaws. Angle Orthod.

[30] Al-Moghrabi D, Salazar FC, Pandis N, Fleming PS (2017). Compliance with removable orthodontic appliances and adjuncts A systematic review and meta-analysis. Am J Orthod Dentofacial Orthop.

[31] Berkowitz S (2013). Cleft Lip and Palate: Diagnosis and Management.

[32] Kravitz ND (2014). Treatment with the mandibular Arnold expander. J Clin Orthod.

[33] Miner RM, Al Qabandi S, Rigali PH, Will LA (2012). Cone-beam computed tomography transverse analysis Part I: Normative data. Am J Orthod Dentofacial Orthop.

[34] Tai K, Park JH (2010). Dental and skeletal changes in the upper and lower jaws after treatment with Schwarz appliances using cone-beam computed tomography. J Clin Pediatr Dent.

[35] Raucci G, Pachêco-Pereira C, Elyasi M, d'Apuzzo F, Flores-Mir C, Perillo L (2016). Short- and long-term evaluation of mandibular dental arch dimensional changes in patients treated with a lip bumper during mixed dentition followed by fixed appliances. Angle Orthod.

[36] Vargo J, Buschang PH, Boley JC, English JD, Behrents RG, Owen 3rd AH (2007). Treatment effects and short-term relapse of maxillomandibular expansion during the early to mid mixed dentition. Am J Orthod Dentofacial Orthop.

[37] Ferris T, Alexander RG, Boley J, Buschang PH (2005). Long-term stability of combined rapid palatal expansion-lip bumper therapy followed by full fixed appliances. Am J Orthod Dentofacial Orthop.

[38] Little RM, Riedel RA, Stein A (1990). Mandibular arch length increase during the mixed dentition postretention evaluation of stability and relapse. Am J Orthod Dentofacial Orthop.

[39] Osborn WS, Nanda RS, Currier GF (1991). Mandibular arch perimeter changes with lip bumper treatment. Am J Orthod Dentofacial Orthop.

[40] Fränkel R (1974). Decrowding during eruption under the screening influence of vestibular shields. Am J Orthod.

[41] Gurgel JA, Pinzan-Vercelino CRM, Leon-Salazar V (2017). Maxillary and mandibular dentoalveolar expansion with an auxiliary beta-titanium arch. Am J Orthod Dentofacial Orthop.

[42] McNamara JA, Baccetti T, Franchi L, Herberger TA (2003). Rapid maxillary expansion followed by fixed appliances a long-term evaluation of changes in arch dimensions. Angle Orthod.

[43] Steiner GG, Pearson JK, Ainamo J (1981). Changes of the marginal periodontium as a result of labial tooth movement in monkeys. J Periodontol.

[44] Thilander B, Nyman S, Karring T, Magnusson I (1983). Bone regeneration in alveolar bone dehiscences related to orthodontic tooth movements. Eur J Orthod.

[45] Morris JW, Campbell PM, Tadlock LP, Boley J, Buschang PH (2017). Prevalence of gingival recession after orthodontic tooth movements. Am J Orthod Dentofacial Orthop.

[46] Pratelli P, Gelbier S, Gibbons DE (1998). Parental perceptions and attitudes on orthodontic care. Br J Orthod.

[47] Sarul M, Lewandowska B, Kawala B, Kozanecka A, Antoszewska-Smith J (2017). Objectively measured patient cooperation during early orthodontic treatment Does psychology have an impact?. Adv Clin Exp Med.

